# ICEPO: the ion channel electrophysiology ontology

**DOI:** 10.1093/database/baw017

**Published:** 2016-04-06

**Authors:** V. Hinard, A. Britan, J.S. Rougier, A. Bairoch, H. Abriel, P. Gaudet

**Affiliations:** ^1^CALIPHO Group, SIB Swiss Institute of Bioinformatics, 1 rue Michel-Servet, CH-1211 Geneva 4, Switzerland; ^2^University of Bern, Murtenstrasse 35, CH-3008 Bern, Switzerland and; ^3^Department of Human Protein Science, University of Geneva Medical School, 1 rue Michel-Servet, CH-1211 Geneva 4, Switzerland

## Abstract

Ion channels are transmembrane proteins that selectively allow ions to flow across the plasma membrane and play key roles in diverse biological processes. A multitude of diseases, called channelopathies, such as epilepsies, muscle paralysis, pain syndromes, cardiac arrhythmias or hypoglycemia are due to ion channel mutations. A wide corpus of literature is available on ion channels, covering both their functions and their roles in disease. The research community needs to access this data in a user-friendly, yet systematic manner. However, extraction and integration of this increasing amount of data have been proven to be difficult because of the lack of a standardized vocabulary that describes the properties of ion channels at the molecular level. To address this, we have developed Ion Channel ElectroPhysiology Ontology (ICEPO), an ontology that allows one to annotate the electrophysiological parameters of the voltage-gated class of ion channels. This ontology is based on a three-state model of ion channel gating describing the three conformations/states that an ion channel can adopt: closed, open and inactivated. This ontology supports the capture of voltage-gated ion channel electrophysiological data from the literature in a structured manner and thus enables other applications such as querying and reasoning tools. Here, we present ICEPO (ICEPO ftp site: ftp://ftp.nextprot.org/pub/current_release/controlled_vocabularies/), as well as examples of its use.

## Introduction

Ion channels are pore-forming transmembrane proteins that selectively allow ions to flow across the plasma membrane according to electro-chemical gradients. They play key roles in diverse cellular processes, including nerve and muscle excitation, synaptic transmission, cardiovascular regulation, hormone secretion and sensory transduction. In humans, there are 344 genes encoding ion channels (1) and mutations in > 126 of these genes have been associated with diseases (https://search.nextprot.org/proteins/search?mode=advanced & queryId= NXQ_00208).

Disruption of any aspect of ion channel function can cause a wide spectrum of diseases, known as channelopathies. Approximately 160 human diseases resulting from mutations in ion channels have been identified (1). Channelopathies can affect the nervous, cardiovascular, respiratory, endocrine, urinary and immune systems. Moreover, ion channel malfunction is suspected to have a role in the pathogenesis of cancer, gastrointestinal or psychiatric disorders (2). In addition, ion channels are the targets of a myriad of drugs that are used in many clinical indications.

### Classes of ion channels

Ion channels can be classified according to either (i) the type of ions for which they are permeable, (ii) their three dimensional structure (1) or (iii) the type of stimulus that triggers their activation gating. The stimulus-gated classification can be further sub-divided based on the specific stimulus that triggers their activation: changes in membrane potential (or voltage), ligands, temperature, light and by the stretching or deformation of the cell membrane (3, 4).

Ion channels that open following a change in the membrane voltage potential are known as ‘voltage-gated ion channels’ (5). ‘Ligand-gated ion channels’ allow ions to flow across the pore in response to the binding of a chemical messenger (ligand) to the cytoplasmic or extracellular side of the channel (6). These two families are the most important ones, with ∼100 proteins each in human. ‘Temperature-gated ion channels’ are represented by thermosensitive ion channels that belong to the Transient Receptor Potential channel family. They allow animals to sense hot and cold environment and react in a suitable manner. The only known natural ‘light-activated ion channels’ are found in green algae and are named channelrhodopsin-1 and -2 (7). Finally, the ‘mechanically gated ion channels’ are ion pore-forming proteins able to detect mechanical stimulation such as tension, pressure, stretch and cell volume change. Following membrane deformation, they open and let ions pass triggering an appropriate electrochemical response to the stimulus. There are only five genes in human whose product displays mechanically gated properties but several other types of ion channels such as the ligand-gated NMDA receptors or ENaC proteins can be activated by membrane deflection (8).

The ontology we present focuses on the description of the biophysical properties of voltage-gated ion channels. We limited our scope on this class because it is one of the largest, and the most important one with respect to the number of genes associated with channelopathies.

### Voltage-gated ion channel gating

**Figure 1.**
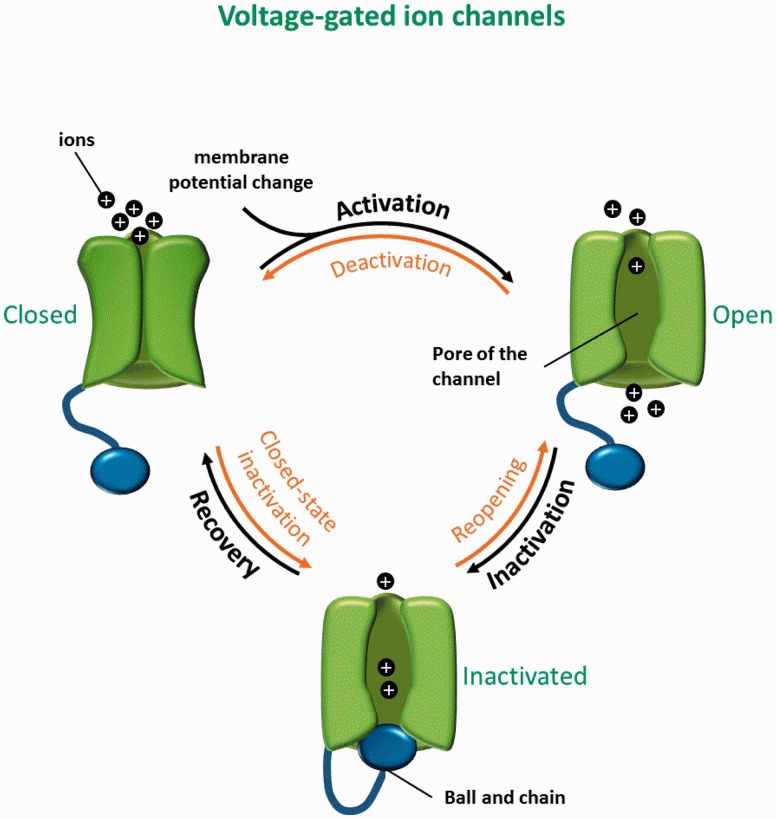
Three-state model of voltage-gated ion channels. Closed, open and inactivated states with the corresponding transitions are shown.

The gating dynamics of the voltage-gated ion channels include three main transitions: opening, inactivation and closing. Opening of the channel pore leads to the flow of ions through protein according to the electro-chemical gradient existing across the membrane. This opening is regulated by the gating of the pore. In response to changes in transmembrane electrical potential difference, ion channels go from a closed state (non-conducting) to an open-state (permeable to ions) as a result of a conformational change in the pore. This transition is referred to ‘activation’. For example, voltage-gated ion channels have a voltage-sensor, consisting of a collection of charged amino acids that move under the influence of the membrane electrical field, thus opening the pore (Figure 1).

Following activation, voltage-gated ion channels go through an inactivated state during which the channel is non-conducting and refractory to open, so-called inactivation (Figure 1). The inactivated state is followed by the return to the closed state via a transition named recovery from inactivation. Once in the closed or resting state, the ion channel can be activated once again and the cycle can resume.

All of these transitions are reversible: the open channel can revert to the closed state, a transition named deactivation; it can go from the inactivated state to the open state, or reopening; and finally, a closed channel can go to the inactivated state, via closed-state inactivation. These transitions only happen when the energetic cost and constants rate are favorable (Figure 1).

### Gating kinetics

While the main factor influencing voltage-gated ion channel gating is the change transmembrane voltage difference, the molecular dynamics are also dependent on the transition rate constants also known as gating kinetics. The gating kinetics are essential in determining the role of each ion channels by controlling the cellular excitability (9). Most of the voltage-gated ion channels exhibit delays in their time course, reflecting multiple sub-transition steps between the main states. For example, after depolarization, voltage-gated ion channels require several sequential steps involving the movement of the gating charge to fully open the activation gate. A higher depolarized potential accelerates those steps by increasing their rate constants (10). Moreover, ion channel kinetics are also dependent on inactivation mechanisms. The open time of an ion channel depends on the time required to close the inactivation gate. For example, voltage-gated potassium channels can be divided in two types according to their inactivation kinetics: the A-type K+ channels being fast inactivating channels and the delayed rectifiers being very slow in their inactivation (11).

### Available ontologies and databases relevant to ion channel function

Approximately 10 000–15 000 original scientific articles are published every year on ion channels, covering topics such as their molecular and cellular characterization, their pathophysiological roles, as well as new strategies to treat and/or prevent diseases (12). Like many other genes, the genes encoding channel subunits have been found to show an impressive polymorphism. Disease-causing genetic variants can affect any aspect of the molecular mechanisms of ion channel activity. To develop a tool to classify, analyse and make predictions about ion channel variants, it is necessary to precisely describe these defects in a structured data model. We first investigated available resources that describe the biophysical parameters of ion channels.

Gene Ontology (GO) (13), the most widely used controlled vocabulary in biology, has terms for all classes of ion channels: voltage-gated ion channel activity (GO:0005244), ligand-gated ion channel activity (GO:0015276), temperature-gated ion channel activity (GO:0097603), light-activated ion channel activity (GO:0010461) and mechanically gated ion channel activity (GO:0008381). It has also specific terms for biological processes such as the trafficking or the clustering of proteins at the plasma membrane. GO molecular function terms describe activities that occur at the molecular level, such as ‘catalytic activity’ or ‘binding activity’. However, GO does not dwell on the details of the biophysical properties of proteins. As explained in the documentation (http://geneontology.org/page/molecular-function-ontology-guidelines), the description of reactions as GO functions does not split each step of the reaction in different functions that would describe the atomic or subatomic terms; rather, it considers the starting state and the end state in terms of the molecules involved. Thus, the level of granularity of GO functions is not precise enough to capture biophysical properties of ion channels, such as transition states. The other types of biological processes impacting the ion channel function being already described in GO, it is out of the scope of the ICEPO.

NIF (Neuroscience Information Framework), an initiative of the NIH Blueprint (http://neuinfo.org/about/index.shtm), groups nearly all the resources covering the current databases, ontologies and terminologies related to neurosciences. One of the main resources supported by NIF that caters for neuroscience ontologies is the neuroscience lexicon: NeuroLex (http://neurolex.org/wiki/Main_Page). This resource covers all the terminologies about neuronal cell types, brain regions, related diseases, electrophysiological protocols and biological processes, all organized in a consistent hierarchy (14). Channelpedia (http://channelpedia.epfl.ch/) is a database developed by the Blue Brain Project and contains an extensive and comprehensive amount of ion channel information captured in research articles (15). IUPHAR, the International Union of basic and clinical PHARmacology (http://www.guidetopharmacology.org/) focuses on pharmacological targets and drugs acting on these targets. It captures ion channel features including some functional and biophysical characteristics, as well as several clinically relevant mutations (16).

However, none of the above-mentioned resources define the sub-states of ion channel gating in sufficient details to capture electrophysiological experiments performed in this field. In order to address this gap, we have started to develop a new ontology describing the biophysical properties of ion channels: the Ion Channel ElectroPhysiology Ontology (ICEPO). ICEPO focuses on the biophysical parameters describing the gating properties of ion channels and more precisely the sequential transitions between their different gating states. The goal is to provide a standardized and open ontology that enables the description of voltage-gated ion channel molecular function and the phenotypic effects of ion channel variants.

## Materials and Methods

### Development of ICEPO

ICEPO was developed using OBO-Edit. Terms are linked by the relations ‘is_a’ and ‘part_of’, and is organized as a simple hierarchy, where each term has a single parent.

**Figure 2.**
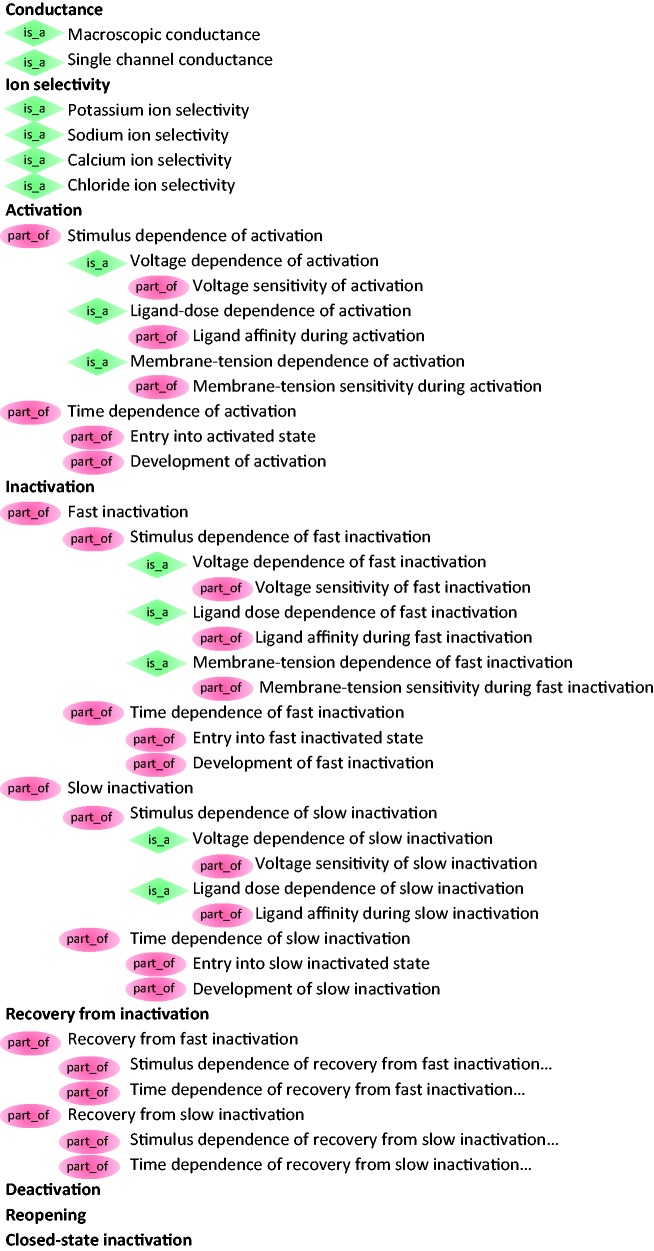
The ICEPO.

The structure of ICEPO is based on (i) the two main parameters of ion channels during their activity: the conductance and the ion selectivity and (ii) the six possible molecular transitions through which they can go: activation, inactivation, recovery, deactivation, reopening and closed-state inactivation. In addition, each transition classes are further divided into sub-classes that capture the two main factors affecting each state: the stimulus and the time. ICEPO currently contains 48 terms (Figure 2). The higher level terms of ICEPO are considered children of the GO concept ion channel activity (GO:0005216), which makes ICEPO easily interoperable with GO.

### The main concepts in ICEPO

The ‘conductance’ of an ion channel is the degree to which electric current carried by the ions flows through the channel. We differentiated two types of conductance according to the methods of ion channel activity recording: the macroscopic conductance, measured by whole-cell recording, which determines the electric current that flows through a population of channels in a macropatch; and the single channel conductance, measured by single channel recording, which determines the electric current that flows through a single channel in a membrane patch.

The ‘ion selectivity’ is the ability of the channel to be permeable to a single ion (potassium, sodium, calcium…) or one type of ion (cation or anion) while excluding the other. This property depends on the selectivity filter, a region of the pore of the protein with a specific conformation adapted to the ion that can flow through it.

The ‘activation’ is the transition from the closed to the open state. The opening of the channel occurs in response to a gating signal, allowing the transfer of ions through the pore.

The ‘inactivation’ is the transition from the open to the inactivated state. Ion channels have an inactivating gate that closes in the continued presence of the gating signal.

The channel is then in a refractory period. No ion can pass through the pore and it is unresponsive to stimulus (inactivated).

At least two different inactivation processes have been described: fast inactivation and slow inactivation, according to the time ion channels take to enter and remain in the inactivated state (17). However, not every voltage-gated ion channel undergoes both forms of inactivation.

The ‘fast inactivation’ is a rapid type of inactivation happening typically in few milliseconds at the macroscopic level. For voltage-gated sodium channels, the fast inactivation is mediated by a globular region of the protein (so-called ball-and-chain) that enters the pore from the cytoplasmic side and physically prevents ions to pass. This type of fast inactivation occurs only when the channel is open and the membrane is still depolarized.

The ‘slow inactivation’ is a deeper inactivation process that can take place instead or in addition to the fast inactivation. For voltage-gated ion channels, it has been shown that slow inactivation, also known as C-type inactivation, lasts longer than fast inactivation. This delay is induced by conformational changes at the selectivity filter region and the protein requires more time to recover from it.

The ‘recovery from inactivation’ is the transition from the inactivated to the closed state in which the channel is non-conducting. Ion channels can return to this state in absence of stimulus. For the voltage-gated sodium channels, this transition requires the repolarization of the membrane potential in order to shrink the pore and relieve the inactivated gate.

As there are two inactivated states, the fast and the slow, there are two corresponding terms for recovery: recovery from fast inactivation and recovery from slow inactivation.

The ‘deactivation’ is the direct transition from the open to the closed state. This transition occurs at different degrees, or not at all, depending on the type of ion channels. Deactivation rarely happens under physiological conditions for voltage-gated sodium channels that normally inactivate immediately after opening. However, some *SCN4A* variants have been shown to lead to most clinically severe form of paramyotonia congenita because they impact stronger the deactivation rate constants and induce a temperature-dependent hyperexcitability of the muscle cell (18).

The ‘reopening’ is the transition from the inactivated to the open state. This transition results in the so-called resurgent sodium current observed in different types of neurons (19). However, it has also been observed in mutant channels, which allow the inactivated gate to open before the pore has gone through the closed state (20).

The ‘closed-state inactivation’ is the transition from the closed to the inactivated state. This has been extensively studied for voltage-gated potassium channels, such as Kv4.1 (*KCND1*), Kv4.2 (*KCND2*) and Kv4.3 (*KCND3*), which undergo physiologically relevant closed-state inactivation at hyperpolarized membrane potential and less frequent inactivation from open state (21).

### The gating dependencies in ICEPO

To fully define the mechanism of ion channels transitions, ICEPO also describes the characteristics of each molecular transition: the stimulation-dependence (voltage dependence and sensitivity) and the time-dependence (entry into the state and development of the state).

First, each transition depends on the level of stimulation, referred to as ‘stimulation dependence’. The probability of opening of voltage-gated ion channel depends on the voltage difference across the plasma membrane. For most voltage-gated ion channels, the greater the depolarization of the transmembrane voltage, the greater the activation of the ion channels, reflected by the amplitude of the current (amount of ions going through the pores). To describe these properties, electrophysiologists perform steady-state activation and inactivation analysis to estimate the voltage-dependencies of voltage-gated ion channels. For each transition, we described the concept of ‘stimulus dependence’ and created the corresponding child term for each transition: voltage-dependence of activation, voltage- dependence of fast inactivation, voltage-dependence of slow inactivation, etc. In addition, ion channel activity depends on its sensitivity to the stimulus. For example, different voltage-gated ion channels have different sensitivity to voltage. Thus, we created terms to specifically describe the voltage sensitivity for each transition.

Second, each transition has a specific rate constant referred to as ‘time dependence’. The time required for the full activation of voltage-gated ion channels can be measured through time-to-peak analysis in whole-cell recording or latency to first opening in single channel recording. We defined both as the entry into activated state. We defined also the entry into the (fast or slow) inactivated state reflecting the time required for ion channels to be fully inactivated. This parameter is often measured through decay phase or peak-to-baseline analysis. In addition, the time during which ion channels stay in a state can be measured. For example, single channel recording enables to determine how long an ion channel remains open during a stimulation event, the so-called mean open time. We defined it by the development of activation. This type of property can be applied for each transition: development of activation, development of fast inactivation, development of slow inactivation, etc.

### Annotations using ICEPO

ICEPO enables to accurately annotate each step of voltage-gated ion channel activity. Using this ontology, we have started to annotate the family of voltage-gated sodium channels in human, aiming to capture all effects on the channels caused by mutations found in patients. We illustrate how ICEPO is used for annotation using the *SCN5A* (Nav1.5) variant p.Ile141Val. This variant was found in 16 adults from a Finnish family with a history of exercise-induced polymorphic ventricular arrhythmia (22). When expressed in the HEK293 cell line, the characterization of the variant biophysical properties using whole-cell voltage clamp recording protocol showed that this mutation shifted the activation curve toward more negative potentials increasing the window current and hastened the kinetics of both activation and inactivation. This is captured using ICEPO as hyperpolarizing the voltage dependence of activation, as well as hastening the entry into both activated and inactivated states (Table 1). At the cellular level, these changes result in a decrease in the excitability threshold of cardiac cells. On the other hand, a number of other parameters are not affected, including the recovery from inactivation and the slow inactivation steps.

**Table 1.** Effects of the SCN5A-p.Ile141Val mutation on the electrophysiological parameters of the channel

**SCN5A-p.lle141 Val**	Has normal	Macroscopic conductance
	Hyperpolarizes	Voltage dependence of activation
	Has normal	Voltage dependence of fast inactivation
	Has normal	Voltage sensitivity of activation
	Has normal	Voltage sensitivity of fast inactivation
	Hastens	Entry into activated state
	Hastens	Entry into fast inactivated state
	Has normal	Recovery from fast inactivation
	Has normal	Entry into slow inactivated state
	Has normal	Recovery from slow inactivation

The relation ‘has normal’ is used to describe results that do not noticeably differ from wild-type.

Capturing the exact molecular details of the defect of variants will allow to make predictions on possible disease outcomes for new variants once sufficient data has been gathered.

## Conclusion

This study presents the first development of ICEPO, an ontology that describes selected molecular steps of voltage-gated ion channel function. With minor modifications and extensions, the ontology could be used to annotate the electrophysiological parameters of any class of ion channel. We have started to use ICEPO to annotate the phenotypic effects of voltage-gated sodium channel sequence variations. The ontology, as well as our annotations based on the ICEPO will be made available in the human protein-centric knowledgebase neXtProt (http://www.nextprot.org) and freely accessible for both scientists and clinicians. Our main goal is to make use of all pertinent knowledge on the ion channel mutations and on their biophysical properties to help the prediction of the pathogenicity of newly discovered genetic variation.

## Availability

ICEPO is available on the neXtProt ftp site: ftp://ftp.nextprot.org/pub/current_release/controlled_vocabularies/.  Feedback and suggestions can be sent to support@nextprot.org.
